# Modulation of the type and excitation region of plasmonic topological quasiparticles in a metasurface by tailoring the excitation light

**DOI:** 10.1515/nanoph-2025-0200

**Published:** 2025-07-04

**Authors:** Xinru An, Peng Lang, Xuefeng Shi, Boyu Ji, Feng Lin, Yihe Lin, Yang Xu, Xiaowei Song, Jingquan Lin

**Affiliations:** School of Physics, 47819 Changchun University of Science and Technology, Changchun 130022, China; 47819 Zhongshan Institute of Changchun University of Science and Technology, Zhongshan 528400, China; School of Physics, Peking University, Beijing 100871, China; College of Electronic Information and Optical Engineering, Nankai University, Tianjin 300350, China

**Keywords:** plasmonic skyrmions, plasmonic merons, metasurface, geometric phase, scanning near-field optical microscopy

## Abstract

We propose and demonstrate to manipulate the generation of plasmonic skyrmion and meron as well as their field spatial distribution with a metasurface composed of T-shaped nanoslit arrays by tuning the wavelength and spin of the excitation light. Simulation results show that plasmonic skyrmion and meron can be constructed and their field distribution can be manipulated within the proposed metasurface. Controllable unidirectional propagation of surface plasmon polaritons, which depends on the wavelength and spin of the incident circularly polarized light via the geometric phase introduced by the T-shaped nanoslit array, supports the formation of plasmonic topological quasiparticles. Scanning near-field optical microscope (SNOM) is employed to image the field profile of the plasmonic topological quasiparticles under the different excitation conditions, and it is found out that the field profile obtained by SNOM is in good agreement with the simulation result.

## 1 Introduction

Metasurface composed of two-dimensional array of subwavelength meta-atom provides a promising platform for optical field manipulation [1], [2]. Subwavelength structure can control the phase and amplitude of surface plasmon polaritons (SPPs) through various configurations, such as aperture [3], antenna nanostructure [4], and groove [5]. By designing the structural parameter and arrangement of the unit cell in the metasurface, wavefronts of the reflected, transmitted, and surface waves can be almost arbitrarily engineered, facilitating enormous achievements such as super-lens [6], chiroptical reflection [7], and nondiffractive SPPs [8].

Skyrmions and merons are topologically protected quasiparticles discovered in a wide range from high-energy to condensed-matter physics [9], [10]. Recently, plasmonic topological quasiparticles have been generated by surface plasmon polariton (SPP) waves, i.e., plasmonic skyrmions and merons constructed by circularly polarized light illuminating hexagonal structure and quadrilateral structure [11], [12], [13]. Due to the tight confinement of optical field, plasmonic topological quasiparticles are promising for multichannel data parallelization and high-speed information transmission. For optical skyrmion, the orientation of vector field changes progressively from the central upward/downward state to its inverse state at the edge of the lattice [14], [15], [16], [17], [18], [19]. While the orientation of the vector consecutively falls to be parallel to the surface at the edge for a meron with half-integer topological number [20], [21], [22]. As the novel information carriers, switching of their spatial distribution and topological type is crucial for providing the tunable degree of freedom in information encoding [23], [24], [25]. However, flexible manipulation of plasmonic topological quasiparticles through all-optical method remains challenging, limiting their further applications in versatile topological photonic devices.

Taking the advantage of T-shaped nanoslit arrays, we achieve manipulation of the type and excitation region of plasmonic topological quasiparticles by adjusting the wavelength and spin of the incident circularly polarized (CP) light here. Unidirectional propagation of SPPs excited by CP light at the resonant wavelength due to the geometric phase introduced by the etched T-shaped nanoslit arrays provides the basis for the generation of plasmonic topological quasiparticles, and the wavelength-dependent plasmonic topological quasiparticles can be constructed by precisely tuning the structural parameters of the nanoslit arrays arranged as equilateral triangle and quadrilateral shapes. Manipulation of plasmonic topological quasiparticles is further demonstrated experimentally with scanning near-field optical microscopy (SNOM), persisting the excellent switching performance with low crosstalk. This dual-parameter modulation approach would significantly expand the optical information encoding capacity based on the topological photonic structure.

## 2 Design and fabrication

### 2.1 Design of metasurface

Topological property of two-dimensional optical skyrmion can be characterized by the skyrmion number *S* [13]:

(1) $$S=\frac{1}{4\pi }\int \underset{A}{\int }\overset{⇀}{n}\cdot \left(\frac{\partial \;\overset{⇀}{n}}{\partial x}\times \frac{\partial \;\overset{⇀}{n}}{\partial y}\right)\text{d}x\text{d}y$$

where *A* denotes the region of skyrmion lattice, $\overset{⇀}{n}$ represents the unit vector field, e.g., electric field, magnetic field, spin, or Stock vector. For a skyrmion, the skyrmion number *S* is ±1 with the sign dependent on the polarization of the vector field on the center of the lattice. Meanwhile, the skyrmion number *S* is ±1/2 for a meron with the difference of the vector field oriented parallel to the surface at the edge. Plasmonic skyrmions and merons are generated by the superposition of the converging SPP waves excited from boundaries of triangles and squares, which comprise T-shaped nanoslit resonator (NSR) arrays. By controlling the phase difference and amplitude of multiple SPP fields, complex phase distributions can be generated after interference of multiple SPP fields, thus meeting the topological requirements of plasmons skyrmion and meron.

**Figure 1:**
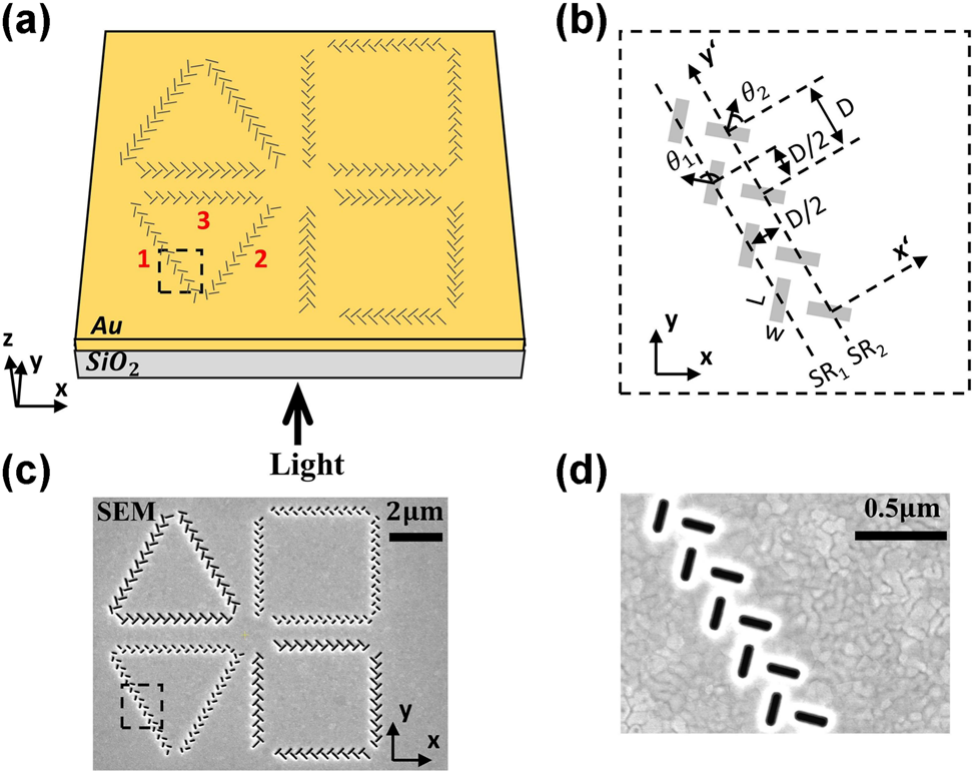
Fabrication of the proposed metasurface. (a) Schematic diagram of the proposed metasurface consisting of fourteen groups of T-shaped nanoslit resonator (NSR) arrays etched on a gold film. The NSR array in the lower-left equilateral triangular is numbered as 1–3. (b) Details of T-shaped nanoslit array. (c) Scanning electron microscope (SEM) images of the fabricated metasurface coupling structure and the detail (d) of the nanoslit array.

T-shaped nanoslit resonator (NSR) arrays with different structural parameters are utilized to achieve the selective excitation of plasmonic skyrmions and merons. The schematic diagram of the designed metasurface is shown in Figure 1(a), which is consisted of fourteen T-shaped NSR arrays arranged as two rows of equilateral triangles and quadrilaterals on the metasurface. Structural parameters of lengths, widths, and spaces of the nanoslits in a single T-shaped NSP are the same for the diagonal polygonal structures. A single T-shaped NSR comprises of two orthogonally oriented naonslits with the same width and length. Taking the equilateral triangle at lower-left of the proposed metasurface as an example, the resonant wavelength of the incident light is 633 nm. It is constructed by three T-shaped nanoslit arrays numbered 1–3 with the detailed parameters shown in Figure 1(b). To achieve the unidirectional launching of SPP, the adjacent T-shaped NSRs are separated with the distance of *D* = *λ*_spp_/2 along *y*′ axis parallel to the side of triangle, and nanoslits in one T-shaped NSR are displaced by half a period (*D*/2 = *λ*_spp_/4) along the *x*′ and *y*′ axis in local coordinate system to reduce the near-field coupling and scattering of SPPs from the adjacent apertures. Parameter of *D* = *λ*_spp_/2 is dependent on SPP wavelength, which are 300 nm and 350 nm for the lower and upper equilateral triangles, respectively. The orientation angles of slit resonators (SRs) of SR_1_ and SR_2_ are *θ*_1_ = 3π/4, *θ*_2_ = π/4 with respect to the local coordinate system. The equilateral triangle and quadrilateral metasurface composed of T-shaped nanoslit resonator (NSR) arrays shown in Figure 1(c) is fabricated on a 200 nm thick gold film deposited on a glass substrate by e-beam evaporation. The metal is completely removed from the areas forming the T-shaped NSR arrays by focused ion beam (FIB) etching technique. Scanning electron microscope (SEM) image of the T-shaped NSR array is shown in Figure 1(d).

### 2.2 Selective excitation of SPP with T-shaped NSR array

**Figure 2:**
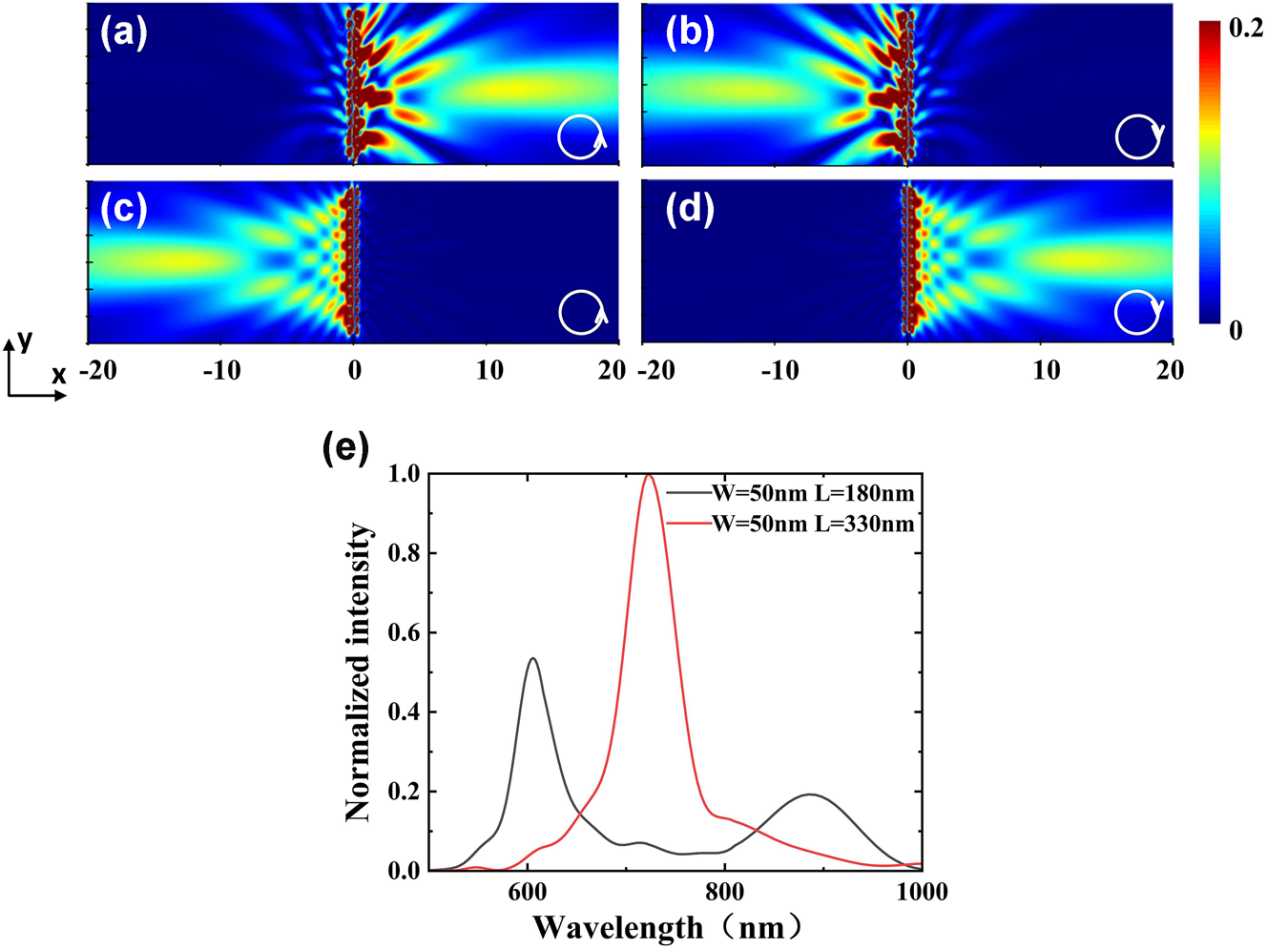
Selective excitation of SPP wave from T-shaped NSR array. (a) and (b) The simulated field profiles of the T-shaped NSR array with *θ*_1_ = 3π/4, *θ*_2_ = π/4 (1st array) under the illuminations of 633 nm LCP and RCP light, respectively. (c) and (d) The simulated field intensity profile of T-shaped NSR array with *θ*_1_ = π/4, *θ*_2_ = 3π/4 (2nd, 3rd arrays) under the illuminations of 633 nm LCP and RCP incident light, respectively. (e) Normalized field intensity versus wavelength for T-shaped NSR array with a constant width of *W* = 50 nm and the different lengths of *L* = 180 nm (black line) and 330 nm (red line).

Under the normal incidence of circularly polarized light, the SPP launching from SR_1_ and SR_2_ has an initial phase difference of *δ* = ±π/2 depending on the spin direction *σ*. Due to the horizontal distance between SR_1_ and SR_2_ is *λ*_spp_/4, there will also be a propagating phase difference of *k*_spp_·*λ*_spp_/4 = π/2 in the direction of either left or right of the SR between the excited SPPs. The initial phase difference of *δ* related to spin and the phase difference of *k*_spp_·*λ*_spp_/4 propagation independent of spin will lead to left or right SPP propagation, where the phase difference of SPP propagation is Δ*φ* = *k*_spp_·*λ*_spp_/4 +*δ*. Under left circularly polarized light incidence, the phase difference between SR_1_ and SR_2_ is Δ*φ*_left_ = *k*_spp_·*λ*_spp_/4 +π/2 for SPP waves. While the phase difference between SR_1_ and SR_2_ is Δ*φ*_right_ = *k*_spp_·*λ*_spp_/4 − π/2 for right circularly polarized light. Therefore, the phase differences for the superimposed SPP propagating to left and right sides of the SR arrays are Δ*φ*_left_ = π and Δ*φ*_right_ = 0. It results in the destructive interference for the exciting SPP launching to the left side of the rectangular side and constructive interference to the right side. The situation is reversed under the excitation of right circularly polarized light. Selective excitation of SPP with the spin of the incident light is verified by illuminating the T-shaped NSR array with CP light using FDTD simulation, and the results are shown in Figure 2(a) and (b), respectively. The propagation directions along −*x* and +*x* directions of the excited SPP waves can be controlled by changing the spin of the incident light. For the 2nd and 3rd arrays, the orientation angles of the SR_1_ and SR_2_ are *θ*_1_ = π/4, *θ*_2_ = 3π/4 with respect to the respective local coordinate system. Therefore, the propagation direction of the excited SPP is along −*x* and +*x* directions with LCP and RCP, respectively, which are exactly opposite to these of the 1st array with the same CP light as shown in Figure 2(c) and (d).

Near field response of the two T-shaped NSR arrays with the different parameters are also investigated using FDTD. Figure 2(e) shows the normalized field intensity spectra for T-shaped NSR array with the constant width of *W* = 50 nm but different lengths of *L* = 180 nm and 330 nm, respectively. The resonant wavelengths are 633 and 722 nm for the NSR arrays with the lengths of *L* = 180 nm and 330 nm, respectively. As shown in the Figure 2(e), the cross-talk between the different NSRs is relatively low. Due to the unidirectional excitation of SPP beams, three SPP beams excited by the side of the constructed equilateral triangle converge to the center and generate plasmonic skyrmion lattices with 633 nm LCP light, similar results can be obtained in upper equilateral triangle with 722 nm LCP light. In the case of equilateral quadrilateral, the generation of plasmonic quasiparticles follows the same rule. The sample is normally illuminated from the substrate side by 633 nm and 722 nm CP laser with the different spins.

### 2.3 Experimental setup

Collection mode scanning near-field optical microscopy (SNOM) (Nanonics Imaging MV-2000) with a gold-coated fiber probe (aperture diameter, 150 nm) is used to map the near-field of the proposed structure. The collection of SPP beam is acquired by an Avalanche Photo Diode (APD) system.

## 3 Manipulation of plasmonic topological quasiparticles

**Figure 3:**
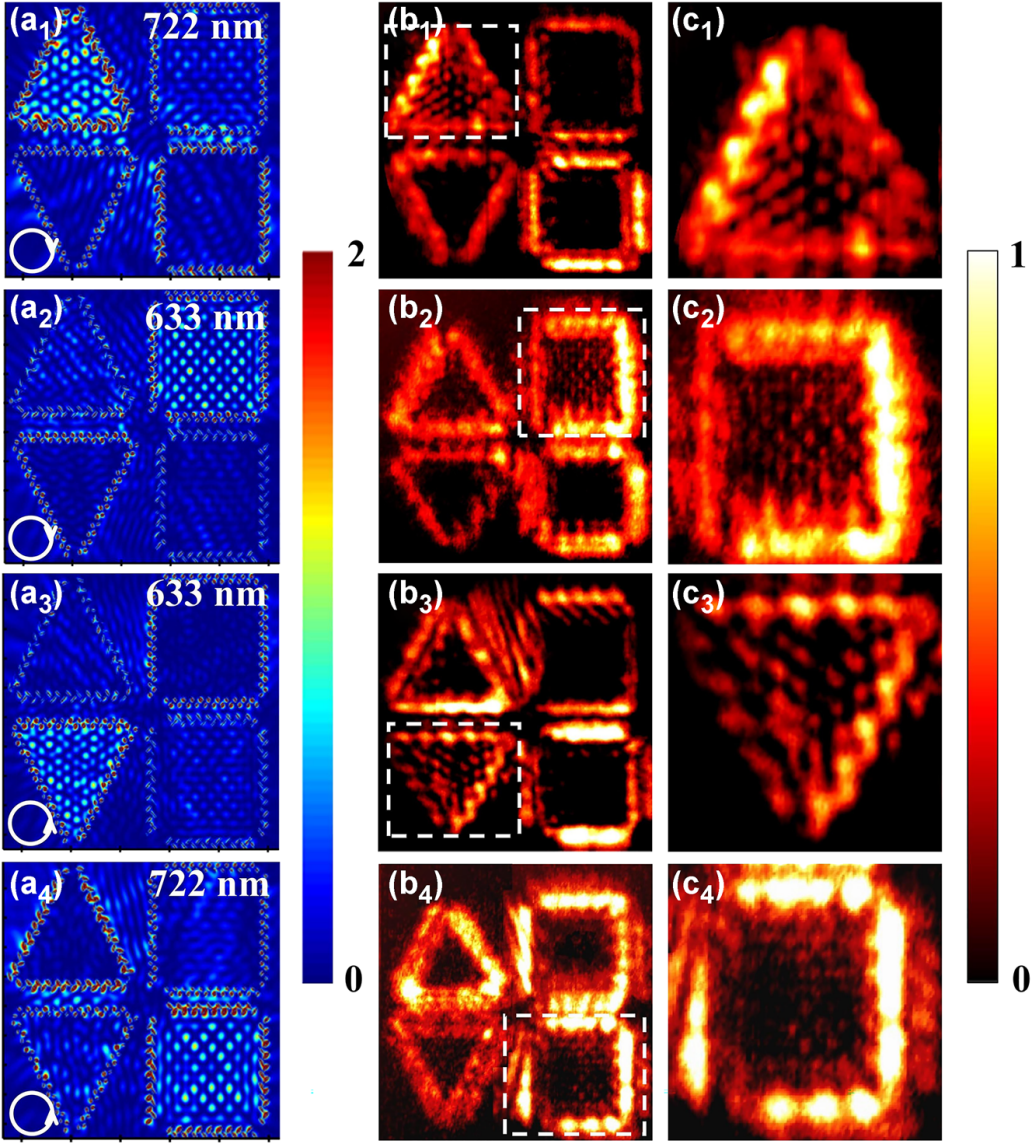
Manipulation of plasmonic skyrmions and merons in the metasruface with FDTD simulation and SNOM measurement. (a) The simulated field profiles of the proposed metasurface. (b) The SNOM images of the proposed metasurface. (c) Zoom-in images of the field profiles extracted in the white dash square in (b). (a_1_), (b_1_), (c_1_) With 722 nm RCP light, (a_2_), (b_2_), (c_2_) with 633 nm RCP light, (a_3_), (b_3_), (c_3_) with 633 nm LCP light, (a_4_), (b_4_), (c_4_) with 722 nm LCP light.

Manipulation of the topological type and region of plasmonic skyrmion and meron is demonstrated with both the numerical simulation and SNOM measurement. Figure 3 presents field profiles of the proposed metasurface under the illumination of circularly polarized light with different wavelengths and spins obtained by FDTD simulation and SNOM measurement. Within the equilateral triangles and quadrilaterals, SPPs can be excited at their sides and converge to construct the topological vector textures with the specific wavelength and spin of the incident CP light. That is, with the illumination of 722 nm RCP (Figure 3(a_1_)) and 633 nm LCP (Figure 3(a_3_)) light, SPPs converge in the centers of the upper and lower equilateral triangles where the plasmonic “hotspots” form skyrmion-like lattices, respectively. Each “hotspot” is surrounded by six “hotspots” with 6-fold rotational symmetry, which is similar to 2D hexagonal crystalline structure. It is the typical character of plasmonic skyrmions [13]. Meanwhile, SPPs converge in the centers of the upper and lower equilateral quadrilaterals with the formation of meron-like lattices by the plasmonic “hotspots” when adjusting the incident light to 633 nm RCP (Figure 3(a_2_)) and 722 nm LCP (Figure 3(a_4_)) light, respectively. In this case, each “hotspots” is surrounded by four “hotspots” with 4-fold rotational symmetry, corresponding to 2D tetragonal crystalline structure as the typical character of plasmonic meron [24].

This modulation behavior of plasmonic topological quasiparticles is further demonstrated with SNOM measurement. The in-plane component *E*_//_ of the electric field can be effectively extracted by the fiber probe of SNOM, whose profiles are closely akin to those of plasmonic skyrmion and meron lattices shown in Figure 3(a_1_)–(a_4_). Figure 3(b_1_) and (b_2_) present the field profiles on the surface of the proposed metasurface with RCP light at the wavelengths of 722 nm and 633 nm obtained by SNOM, respectively. With 722 nm light (Figure 3(b_1_)), the plasmonic skyrmion-like lattices are generated in the center of the upper-left triangular region. When the wavelength of the incident light reduced to 633 nm (Figure 3(b_2_)), the meron-like lattices are produced within the center of upper-right quadrilateral region, which is consistent with the FDTD simulation result. Detail of the field profiles of plasmonic skyrmion and meron lattices extracted from the white dash square in Figure 3(b_1_) and 3(b_2_) are shown in Figure 3(c_1_) and (c_2_). The hexagonal lattices (Figure 3(c_1_)) and tetragonal lattices (Figure 3(c_2_)) can be clearly observed. SNOM measured field profiles on the Au film surface with 633 nm and 722 nm LCP light are shown in Figure 3(b_3_) and (b_4_), respectively. Under 633 nm LCP light (Figure 3(b_3_)), skyrmion-like lattices are generated within the lower-left triangular region. With the wavelength increasing to 722 nm (Figure 3(b_4_)), meron-like lattices are generated within the lower-right quadrilateral region, consistent with the FDTD simulation results. Detail of field profiles of skyrmion lattice and meron lattice extracted from the white dash square in Figure 3(b_3_) and (b_4_) are shown in Figure 3(c_3_) and (c_4_). Figure 3(c_3_) exhibits some irregular morphologies in the skyrmion-like structure while still maintaining 6-fold rotational symmetry. Although the meron intensity in Figure 3(c_4_) is relatively weak, it still demonstrates clear 4-fold rotational symmetry. Notably, there is slight difference between FDTD simulation and SNOM measurement, which can be attributed to the challenges in achieving the precise fabrication of the length, width, distance, and tilt angle of the T-shaped NSR arrays etched on Au film. Deviations in the structural parameters could cause a shift in the resonant wavelength away from the designed wavelength, leading to degrade the field profiles via the resonance crosstalk. The above results show that SNOM measurement results are mainly consistent with FDTD simulation. Manipulation between the plasmonic topological quasiparticles type and excitation region can be achieved by varying wavelengths and spin (*λ* = 633 nm, 722 nm, and *σ* = −1, 1) of the excitation light. This metasurface can also be combined with ultrafast laser pulse, by introducing chirp or the superposition of a delayed orthogonally polarized laser pulse in time domain to adjust the instantaneous wavelength and polarization state [26], [27], achieving active temporal modulation of the types and excitation regions of plasmonic skyrmions and merons.

Table 1 presents the differences between our designed metasurface and the structure that previously used meta-slit arrays, as well as the differences between our designed metasurface and the structure of previously manipulating topological quasiparticles. The comparative analysis can be categorized into two aspects: control degrees of freedom and manipulation content. It can be seen that the manipulation methods of the previous meta-slit array designs and the topological quasiparticle manipulation designs are both single parameter control, and the manipulation content of the topological quasiparticles is also single. And our metasurface provides dual parameters of polarization and wavelength as the degrees of freedom for manipulating the type transformation and position conversion of plasmonic topological quasiparticles. Compared with traditional single degree of freedom modulation methods, this dual-parameter modulation method can significantly improve the information encoding capability, while the topological robustness of skrmions and merons can enhance the anti-interference ability in data transmission. The wavelength-spin multiplexing technology enables multiplexed data encoding, opening new pathways for the development of topological optical devices, particularly in high-density optical storage systems.

**Table 1:** Comparison between metasurface in this work and prior designs.

	Literature	Control degrees of freedom	Manipulation contents
Meta-slit array designs	Lin et al., Science 340(6130), 331–334, 2013. [28]	Polarization only	SPP direction modulation
	Seung-Yeol Lee et al., Optica 2, 6–13, 2015. [29]	Polarization only	Focus direction and position modulation
	Wei et al., Opt. Express 25, 24872, 2017. [30]	Wavelength only	Focus position modulation
Topological quasiparticle manipulation designs	Lin et al., ACS Photonics 8(9), 2567–2572, 2021. [23]	Spatial light modulator	Topological quasiparticles position
	Lin et al., Applied Physics Reviews 11(2), 021408, 2024. [24]	Wavelength only	Topological quasiparticles transformation
	Metasurface in this work	Polarization and wavelength	Topological quasiparticles transformation and position

**Figure 4:**
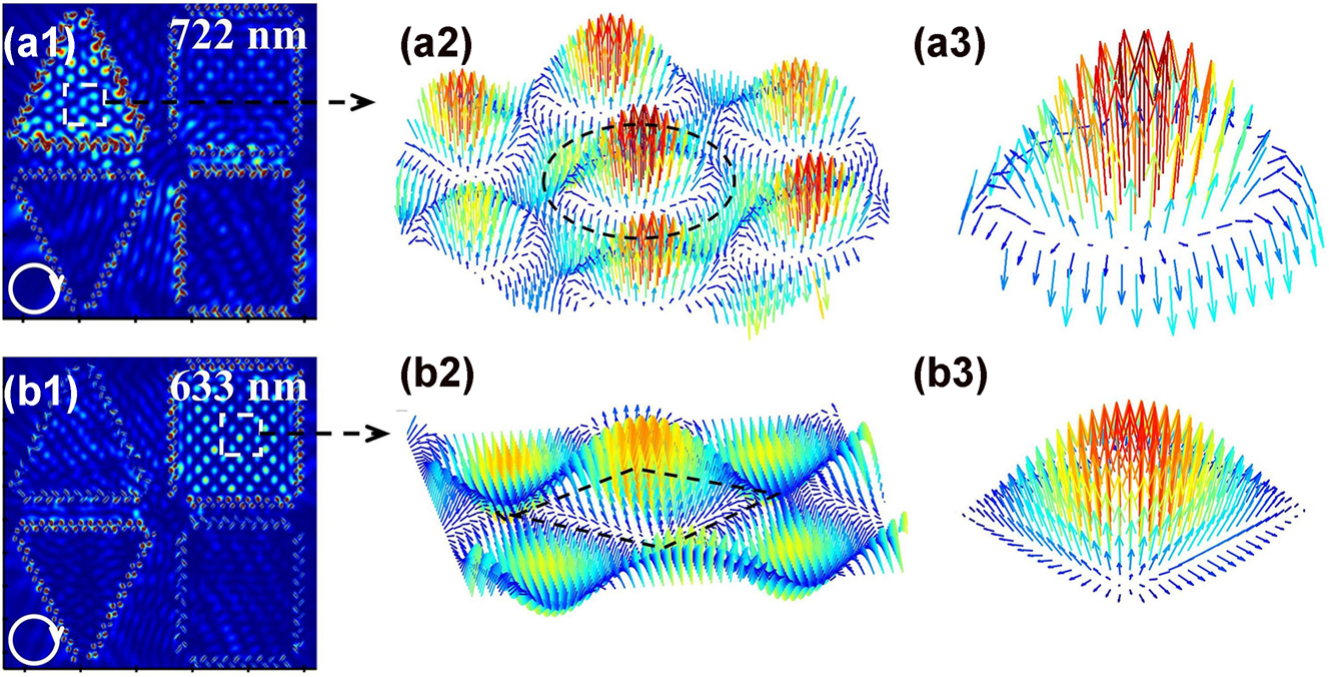
Vector textures of electric field corresponding to the generated plasmonic topological quasiparticles obtained by FDTD simulation. Electric field profiles of plasmonic skyrmions and merons in the proposed metasurface with (a_1_) 722 nm and (b_1_) 633 nm RCP light. Vector texture in the central regions of the upper (a_2_) triangular structure and (b_2_) quadrilateral structures. Details of the vector textures of (a_3_) single skyrmion lattice and (b_3_) single meron lattice extracted in the black dash circle and rhombus in (a_2_) and (b_2_).

To further confirm the skyrmion/meron-like lattices formed in the triangular/quadrilateral regions, vector textures of electric field corresponding to the generated plasmonic topological quasiparticles obtained by FDTD simulation are shown in Figure 4. A three-dimensional electric field vector profile with the square of 1.5 × 1.5 μm^2^ extracted from the central region of the upper-left triangular structure under 722 nm RCP illumination (Figure 4(a_1_)) is illustrated in Figure 4(a_2_). It clearly shows that the vector texture corresponds to plasmonic skyrmion lattice originating from the interference of multiple SPPs, exhibiting a six-fold symmetric distribution of hotspot structure. Detail of vector texture of a single skyrmion lattice extracted from the black dash circle is shown in Figure 4(a_3_). It can be observed that the orientation of the spin vectors gradually changes from the central upward to downward at the edge, and the skyrmion number calculated based on formula (1) is *S*_triangle_ = 0.95, corresponding to optical skyrmion lattice. To verify the emergence of plasmonic meron, the electric field vector texture with the square of 1.5 × 1.5 μm^2^ extracted in the central region of the upper-right quadrilateral structure with 633 nm RCP light (Figure 4(b_1_)) is illustrated in Figure 4(b_2_). The vector texture exhibits the distribution characteristics of plasmonic meron lattice, featuring a fourfold symmetric distribution of hotspot structures. Detail of vector texture of a single meron lattices extracted from the black dash rhombus is shown in Figure 4(b_3_). The orientation directions between the electric field vector at the central and surrounding hotspots are opposite to each other; however, they are identical for the hotspots arranged in the horizontal and perpendicular directions. As a result, it is not possible to generate a closed boundary with the opposite orientations of the electric field from that of the central hotspot in a single meron lattice. It can be observed that the electric field vector pointed upward in the core region and progressively transforms to parallel to the surface at the edges, corresponding to the characteristic pattern of plasmonic meron. The skyrmion number of 0.53 can be obtained for the single plasmonic meron, confirming the formation of plasmonic meron. The texture of the plasmonic field could exhibit oscillation due to the nonzero frequency in time domain, causing the sign of the topological charge to alternate within a femtosecond timescale. In this study, the vector texture is obtained from frequency domain monitor of FDTD solutions, which is Fourier transformation of the time-varying oscillation of the electromagnetic field. As a result, the optical response of oscillating EM field in the defined region can be derived at the specific frequency. In other words, field texture with the topological number of +1 or −1 can be guaranteed at the specific frequencies corresponding to the wavelengths of 722 nm and 633 nm as shown in Figure 4. Meanwhile, the electric field texture would oscillate from +1 to −1 in time domain due to the high frequency of the oscillating field, so the averaging texture is zero. However, the results focus on the topological property of electric field texture in frequency domain where stable topological quasiparticles can be formed.

## 4 Conclusions

In conclusion, manipulation of the type and generation region of plasmonic topological quasiparticles on a metasurface are achieved by tuning the wavelength and spin of CP light. Utilizing composed T-shaped nanoslit array, plasmonic skyrmion and meron can be constructed with the proposed metasurface consisting of two rows of equilateral triangles and quadrilaterals. With RCP light illumination, plasmonic skyrmion lattices generated in the upper-left equilateral triangle is switching into plasmonic meron in the upper-right equilateral quadrilateral by tunning the excitation wavelength from 722 nm to 633 nm, and by increasing the wavelength from 633 nm to 722 nm, switching of the skyrmion in the lower-left region into a meron in the lower-right region can be realized with LCP light illumination. The manipulation function of the designed metasurface is also experimentally demonstrated with SNOM mapping. This work demonstrates that wavelength and spin of the excitation light can be employed as the degrees of freedom for manipulating the type and generation region of plasmonic quasiparticle in the polygonal nanostructure, laying a solid fundamental for information transmission and encoding based on topological photonic structures.
